# Dark-blood Imaging in Coronary CT Angiography using Dual-layer Detector Spectral CT: Effects on Image Quality and Vessel Wall Visibility

**DOI:** 10.2174/0115734056361930251016081026

**Published:** 2025-10-24

**Authors:** Xinglu Li, Xingyu Chen, Wen Chen, ChunHong Hu, Minyi Li, Zhixin Sun

**Affiliations:** 1 Department of Radiology, The Affiliated Changshu Hospital of Nantong University, Changshu No. 2 People’s Hospital, No.18 Taishan Road, Changshu, Jiangsu, 215500, China;; 2 Department of Radiology, The First Affiliated Hospital of Soochow University, No.188 Shizi Road, Suzhou, Jiangsu, 215006, China

**Keywords:** Dual-layer spectral CT, Coronary computed tomography angiography, Subtraction technique, Image quality, Coronary artery disease, Dark-blood images

## Abstract

**Introduction:**

To explore the potential of a newly developed subtraction technique in coronary computed tomography angiography (CCTA) for improving image quality and vessel wall visibility without introducing misregistration artifacts.

**Methods:**

Fifty-six patients who underwent CCTA scans using dual-layer detector spectral CT (SDCT) were retrospectively enrolled. Dark-blood images were generated by subtracting virtual non-contrast (VNC) datasets from 70-keV datasets. Qualitative evaluation of dark-blood images included assessments of image quality, inner-wall visualization, and outer-wall visualization. Quantitative parameters were compared between conventional CCTA images and dark-blood images. The quantitative assessment involved evaluating contrast-to-noise ratio (CNR) and signal-to-noise ratio (SNR). SNR_wall_, SNR_lumen_, SNR_periaortic fat_, CNR_wall-lumen_, and CNR_wall-periaortic fat_ were calculated. Two experienced radiologists independently evaluated the images, and inter-rater variability was assessed.

**Results:**

Patients were categorized into three groups based on plaque types: Group A (calcified plaques, n=88), Group B (non-calcified plaques, n=15), and Group C (vessels without plaque, n=56). Dark-blood images of non-calcified plaques and vessels without plaque exhibited higher image quality and inner-wall visualization scores compared to calcified plaques (all *p* < 0.05). The subjective scores of radiologists showed good consistency (all kappa values > 0.7). Compared to conventional images, dark-blood images demonstrated higher quantitative scores in terms of SNR_wall_, SNR_lumen_, SNR_periaortic fat_, CNR_wall-lumen_, and CNR_wall-periaortic fat_ (all *p* < 0.001).

**Discussion:**

The dark-blood technique enabled superior coronary wall assessment without misregistration artifacts, overcoming a key limitation of prior subtraction CCTA. RCA motion artifacts remain a technical challenge that warrants phase-specific protocol optimization in our study.

**Conclusion:**

Dark-blood images derived from SDCT demonstrated improved image quality of coronary arteries without misregistration artifacts and enhanced visualization of the coronary vessel wall.

## 1 INTRODUCTION

Coronary artery disease (CAD) has been established as the leading cause of death worldwide [1]. Coronary computed tomography angiography (CCTA) plays a pivotal role in the screening, decision-making, and treatment planning of CAD due to its noninvasive nature [2]. However, the interpretation of CCTA may be limited by severe calcified plaques or implanted stents, which induce blooming artifacts, beam-hardening effects, and partial volume averaging due to high intravascular contrast concentrations. These artifacts can impair the image quality of conventional CCTA and lead to an overestimation of stenosis [3]. Additionally, traditional bright-blood images have limitations in visualizing the vessel wall due to low contrast between the arterial wall and the lumen.

Subtraction CCTA was introduced a few years ago as a novel post-processing technique. However, a two-breath-hold subtraction method, which subtracts non-contrast images from contrast-enhanced images, can produce misregistration artifacts [4]. These artifacts have limited the development of subtraction technology. Using a dual-layer spectral detector CT (SDCT), dark-blood images can be generated without misregistration artifacts, because virtual non-contrast (VNC) and virtual monoenergetic images (VMI) are derived from the same dataset in the same cardiac phase.

SDCT uses a single X-ray source paired with a dual-layer detector system that enables spectral separation at the detector level. During image acquisition, the two detector layers simultaneously capture different X-ray absorption profiles, with the low-energy spectrum in the upper layer and high-energy photons in the deeper layer. By linearly combining these signals based on photoelectric and Compton effects, VNC and VMI datasets can be generated [5]. The system provides a range of energy levels from 40 to 200 kilo-electron-volts (keV) [6]. The 70-keV image, which closely approximates a conventional CT image, offers optimal image quality [7, 8], while the VNC image provides diagnostic confidence equivalent to a true non-contrast image [9]. In this study, dark-blood images were generated by subtracting VNC datasets from 70-keV datasets, ensuring complete spatial and temporal alignment. This approach suppressed the contrast agent in the vascular lumen, thereby enhancing lumen-to-wall contrast and facilitating vessel wall evaluation [10].

2. In previous studies, researchers primarily focused on subtracting calcified plaques and stents, often neglecting non-calcified plaques. This study aims to investigate whether a newly developed subtraction technique can enhance coronary vessel wall visualization across various plaque types without causing misregistration, potentially providing insights for the clinical application of subtraction methods.

## 2 MATERIALS AND METHODS

### 2.1 Patient Population

This study was conducted in accordance with the Declaration of Helsinki. The ethics committee of the Affiliated Changshu Hospital of Nantong University approved this single-center retrospective study (CSEY2021115), and the requirement for written informed consent was waived. We included 64 consecutive patients who underwent clinically indicated CCTA using SDCT between February 2021 and October 2022. Patients with implanted stents (n = 3) or those exhibiting motion or pulsation artifacts (n = 5) were excluded. A prospective power analysis (α = 0.05, β = 0.2, effect size = 0.8) indicated a minimum requirement of 51 patients. Ultimately, 56 patients were evaluated. The flowchart of patient enrollment is shown in Fig. (**1**).

### 2.2 Scan Protocol

CCTA was performed using a 64-slice single-source SDCT (IQon, Philips Healthcare). Patients without contraindications and with a heart rate >75 bpm were administered a cardioselective beta-blocker (metoprolol 50 mg) 1 hour prior to scanning. Scans were performed during an inspiratory breath-hold with retrospective electrocardiogram gating in the cranial-caudal direction, covering the entire heart from the carina to the diaphragm. A contrast-enhanced scan was acquired following a non-contrast scan. Iodinated contrast medium (Iopamidol-370, Bayer Schering Pharma) was administered at 1.0–1.2 mL/kg at a rate of 3.5–4.5 mL/s, followed by 30 mL of physiological saline injected at the same rate. An automatic bolus-tracking technique with a trigger threshold of 120–150 Hounsfield units (HU) in the ascending aorta was employed. Scanning parameters were as follows: tube voltage, 120 kVp; tube current, automatically adjusted; collimation, 64 × 0.625 mm; pitch, 0.2; rotation time, 0.27 s; field of view, 220 mm; image matrix, 512 × 512; slice thickness, 0.67 mm; and slice increment, 0.34 mm.

### 2.3 Radiation Dose

The volume of CT dose index (CTDIvol, mGy) and dose length product (DLP, mGy × cm) were automatically recorded post-scanning. The effective radiation dose (ED) was calculated using the formula ED = DLP × 0.014 mSv × mGy^−1^ × cm^−1^ [11].

### 2.4 Image Reconstruction and Post-Processing

**Fig. (2a-l):**
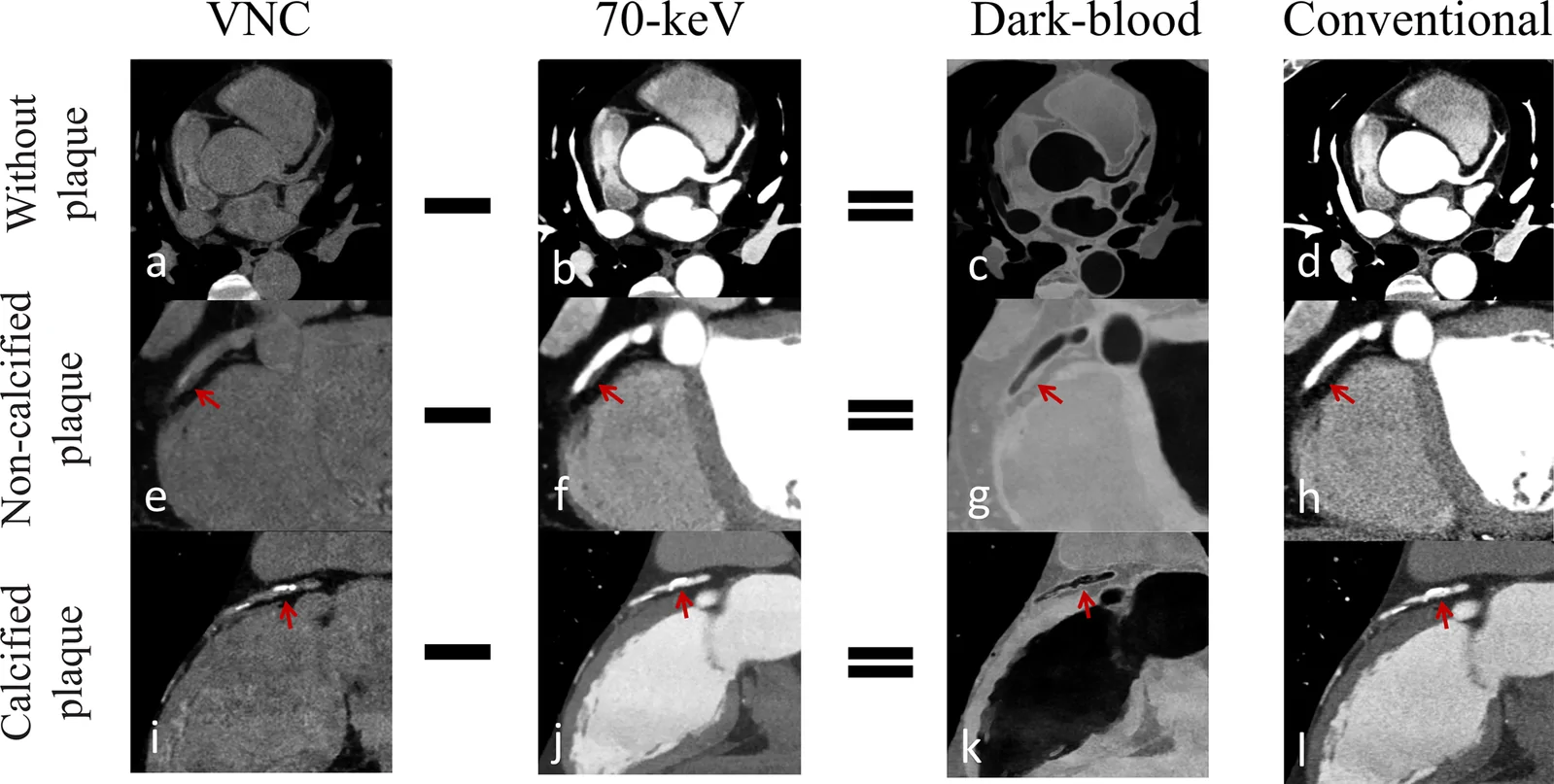
Comparison of different types of plaque between conventional images and dark-blood images. Dark-blood images were generated by subtracting VNC datasets from 70-keV datasets. Vessel without plaque in LAD (**a**-**d**) clearly delineates the inner and outer vessel wall in dark-blood image. The presence of non-calcified plaques in RCA (**e**-**h**) and a calcified plaque in LAD (**i**-**l**) was observed. Compared with conventional images, both calcified and non-calcified plaques can improve the visualization of the vessel wall in dark-blood images.

Images were reconstructed and processed on a post-processing workstation (iDose4, IntelliSpace Portal version 9.0, Philips Healthcare). From a single spectral dataset covering the same range in the same cardiac phase, 70-keV and VNC images were extracted. Dark-blood images were then generated by subtracting the VNC datasets from the 70-keV datasets (Fig. **2a**–**l**), effectively removing the contrast agent from the vessel lumen.

### 2.5 Image Analysis

Segments with a diameter of 1.5 mm or greater were assessed, including the right coronary artery (RCA), left anterior descending artery (LAD), and left circumflex artery (LCX). Plaques were classified as calcified if HU was greater than 350 HU or non-calcified if HU was less than 350 HU.

Conventional and dark-blood images were evaluated blindly by two radiologists with 5 years (XY.C) and 10 years (XL.L) of experience in cardiovascular radiology. Qualitative image analysis of dark-blood images, including image quality, inner-wall visualization, and outer-wall visualization, was independently rated by both radiologists. These characteristics were scored using a 5-point Likert scale. A score of 1 indicated poor image quality with severe noise and artifacts, 2 indicated weak image quality with high noise and minimal artifacts, 3 indicated fair image quality with moderate noise and mild artifacts, 4 indicated good image quality with slight noise, and 5 indicated excellent image quality with sharp edges and no artifacts.

Quantitative image analysis included measurements of signal-to-noise ratio (SNR) and contrast-to-noise ratio (CNR). The mean CT value and standard deviation (SD) were measured three times within a region of interest (ROI) and averaged. ROIs were drawn on the vessel lumen, vessel wall, and periaortic fat near the plaque in the three coronary branches (RCA, LAD, and LCX). For vessels without plaques, ROIs were drawn in the same manner. ROIs were circular with an area of 0.4 mm^2^ and carefully positioned to avoid adjacent tissue and plaque. The size and location of the ROIs were kept consistent across both datasets using copy and paste commands.

SNR_wall_, SNR_lumen_, SNR_periaortic fat_, CNR between the vessel wall and lumen (CNR_wall-lumen_), and between the wall and the periaortic fat (CNR_wall-periaortic fat_) were calculated using the following formula:


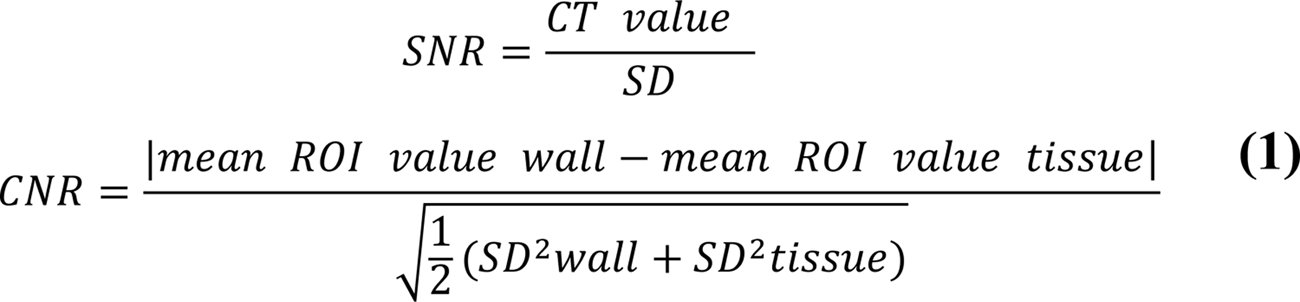


where the subscript “tissue” refers to mean ROI values in the vessel lumen and periaortic fat, respectively.

## 3 STATISTICAL ANALYSIS

Satistical analyses were performed using SPSS statistical software (Version 26.0, IBM, USA). Continuous data were assessed for normality using the Kolmogorov-Smirnov test. Parameters are presented as mean ± standard deviation (SD) for normally distributed data or as counts with percentages for categorical data. For multiple comparisons, paired sample t-tests with Bonferroni correction were applied. Inter-observer agreement for subjective image quality was evaluated using Kappa statistics, with values greater than 0.75 considered excellent, between 0.40 and 0.75 considered fair to good, and less than 0.40 considered poor. The Friedman test was used to compare quantitative parameters between calcified and non-calcified plaques in dark-blood images. A p-value of less than 0.05 was considered statistically significant.

## 4 RESULTS

### 4.1 Participants and Radiation Dose

A total of 56 patients (mean age 65.2 ± 11.4 years, range 36-86 years, 64.3% male) were enrolled. Nine vessels were excluded: six due to motion or pulsation artifacts, three due to diameters less than 1.5 mm, and one due to absence. Finally, 159 vessels [RCA (n=50), LAD (n=56), and LCX (n=53)] were included in the analysis. The vessels were divided into three groups based on plaque type: Group A (calcified plaque, n=88), Group B (non-calcified plaque, n=15), and Group C (vessels without plaque, n=56). Patient characteristics are listed in Table **1**. The CTDIvol, DLP, and ED were 45.97 ± 1.98 mGy, 798.96 ± 72.82 mGy × cm, and 11.19 ± 1.02 mSv, respectively.

### 4.2 Qualitative Evaluation of Image Quality of Dark-blood Images

Two typical examples of calcified and non-calcified plaques in conventional and dark-blood images are shown in Fig. (**2**). Dark-blood images provided improved visualization of the vessel wall, whereas conventional images did not allow for observation of the coronary vessel wall and were therefore not scored. Qualitative evaluation included image quality, inner-wall visualization, and outer-wall visualization. The results of the qualitative evaluation scores for different plaque types are presented in Table **2**.

For all segments, inter-observer agreement was excellent for inner-wall visualization (kappa = 0.76), outer-wall visualization (kappa = 0.72), and overall image quality (kappa = 0.79). Non-calcified plaque images and vessels without plaque had higher qualitative scores for image quality and inner-wall visualization compared to calcified plaque images (all *p* < 0.05). No significant differences were observed between non-calcified plaque images and vessels without plaque. Regarding outer-wall visualization, vessels without plaque had higher qualitative scores (*p* < 0.001), with no significant difference between non-calcified and calcified plaque images.

### 4.3 Quantitative Evaluation of Image Quality

SNR_wall_, SNR_lumen_, SNR_periaortic fat_, CNR_wall-lumen_, and CNR_wall-periaortic fat_ were calculated. The detailed results for SNRs and CNRs in conventional and dark-blood images are presented in Table **3**. The SNRs and CNRs for global segments, as well as for calcified and non-calcified plaques in dark-blood images, were significantly higher than those in conventional images (all *p* < 0.05). The exception was the CNR_wall-lumen_ for non-calcified plaques, where the difference was not statistically significant (*p* = 0.371).

Differences between calcified and non-calcified plaques in dark-blood images are illustrated in Fig. (**3**). SNR_wall_ for non-calcified plaques was significantly higher than that for calcified plaques (*p* < 0.05). However, the differences in other measurements were not statistically significant (*p* > 0.05).

## 5 DISCUSSION

In our study, we investigated a novel subtraction technique based on SDCT, which improved visualization of the coronary vessel wall by suppressing lumen contrast enhancement. All image subtractions were successfully performed without misregistration artifacts. The quality of dark-blood images was sufficient for assessment, with clear depiction of both the inner and outer vessel walls.

Our results indicated that, in dark-blood images, calcified plaques primarily affected overall image quality and inner-wall visualization. However, outer-wall visualization did not show a statistically significant difference compared to vessels without plaques, regardless of plaque type. Additionally, SNR_wall_ in calcified plaques was significantly lower than in non-calcified plaques, which may be due to the limited number of non-calcified plaques included in the study. Compared with conventional images, dark-blood images significantly increased SNRs and CNRs, particularly for calcified plaques, although no significant difference was observed in SNR_wall-lumen_ for non-calcified plaques (*p* = 0.371).

Despite advancements in CT technology, accurately evaluating the percent diameter stenosis of coronary arteries remains challenging. Calcified plaques obscure lumen patency due to partial volume effects and beam-hardening artifacts, often leading to an overestimation of stenosis severity [12]. Moreover, diagnostic accuracy improves with physician experience, emphasizing the importance of proficiency in visual assessment techniques [13]. When the coronary vessel wall is clearly visualized, quantitative assessment of stenosis can substantially reduce inter-physician variability, thereby enhancing the reliability of diagnostic conclusions, as confirmed in our previous studies [14]. Similarly, prior research has demonstrated that CE-boost–derived dark-blood imaging improves image quality and plaque visibility in head and neck CTA [15].

The dark-blood sequence in magnetic resonance imaging has the potential to depict abnormalities in vascular walls [16]. CT post-processing techniques can also achieve blood signal suppression, including dual-energy CT material decomposition [9], manual adjustment of CT value distribution [17], and deep learning reconstruction algorithms [18]. Previous studies primarily focused on assessing calcified plaques and stents. In this study, we explored the value of dark-blood imaging for visualizing vascular walls and investigated differences across various plaque types. Furthermore, subtraction techniques have shown promise in eliminating blooming artifacts [19, 20]. Previous research reported misregistration artifacts in approximately half of the target segments [4], which hindered the development of subtraction techniques. This problem may result from differences in breath-hold times between non-contrast and contrast scans. Kidoh *et al*. developed a technique involving an initial scan at 50 HU attenuation of the ascending aorta followed by a second scan at peak attenuation, which shortened breath-hold time and reduced misregistration artifacts [21]. However, this study included only five patients, and breath-hold times remained longer than in standard scanning protocols. Subtraction using dual-energy CT has also been reported to mitigate mismatching artifacts [22]. In our study, dark-blood images were generated by subtracting virtual non-contrast images from monochromatic images derived from SDCT, captured in the same cardiac phase and image range. This approach enabled iodine subtraction from the lumen while avoiding misregistration artifacts.

In our study, iodine in the lumen was effectively subtracted using the ratio of CT attenuation between VNC and 70-keV images. Xu *et al*. suggested that 100 keV is the optimal monoenergetic energy level for evaluating calcification [23], which is higher than the 90 keV proposed by Stehli *et al*. [24]. Lower energy levels generally enhance intraluminal contrast but increase image noise and artifacts, whereas higher energy levels reduce contrast while decreasing noise and artifacts. The optimal keV threshold for diagnosing calcium-induced coronary stenosis remains debated. In our study, the 70-keV image provided image quality comparable to or better than conventional images [25], and was therefore selected for the subtraction process. VNC images were reconstructed using material decomposition algorithms, which effectively separate iodine from soft tissue. Both VNC and 70-keV images were obtained from routine clinical imaging, and because scan parameters were consistent, the radiation dose was equivalent to that of standard clinical CCTA.

This study has several limitations. First, a significant number of right coronary arteries (RCAs) were excluded due to artifacts. Coronary arteries are often best visualized at different phases of the R-R interval (RCA, 40%; LCX, 50%; LAD, 60–70%) [26]. In our study, reconstruction phases were primarily set at 70% of the R-R interval, which was optimal for the LAD but less so for the RCA. Additionally, the RCA’s location along the atrioventricular groove results in greater motion displacement and artifacts caused by atrioventricular motion asynchrony, which may explain the higher frequency of misregistration. Second, the sample size of this single-center study was relatively small, which may introduce selection bias. Third, plaque visibility was not further assessed. Future comparative studies between dark-blood CTA and MRI could provide a more comprehensive evaluation of coronary plaques.

## CONCLUSION

Our study demonstrates that the subtraction-based dark-blood CT technique using SDCT, which involves subtracting VNC from monochromatic images, effectively eliminates misregistration artifacts while significantly enhancing the visualization of coronary artery walls. The single-phase acquisition avoids temporal mismatches and motion-related artifacts inherent in conventional dual-phase approaches, markedly improving the technical success rate of dark-blood imaging. By suppressing lumen density, this method enhances vessel wall conspicuity without compromising diagnostic image quality and has the potential to serve as a valuable quantitative tool for evaluating coronary stenosis. The observed differential effects of calcified plaques on dark-blood image quality may have clinical implications for treatment stratification. However, this technical feasibility study was not designed to assess therapeutic outcomes, and further research in larger cohorts is warranted. With additional refinement, dark-blood CTA could emerge as a practical, non-invasive alternative for comprehensive coronary artery evaluation.

## Figures and Tables

**Fig. (1):**
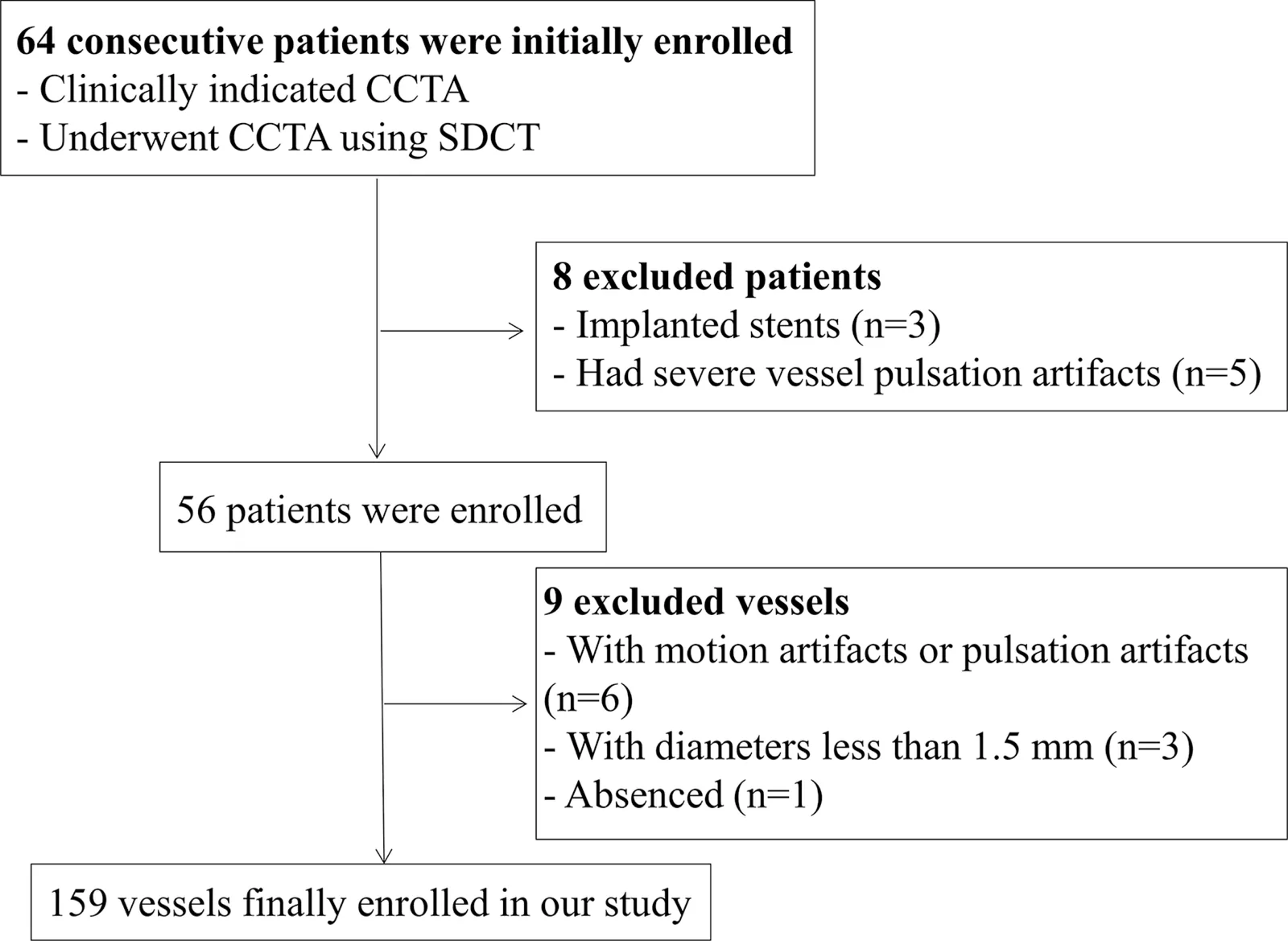
Flowchart of patient enrollment.

**Fig. (3):**
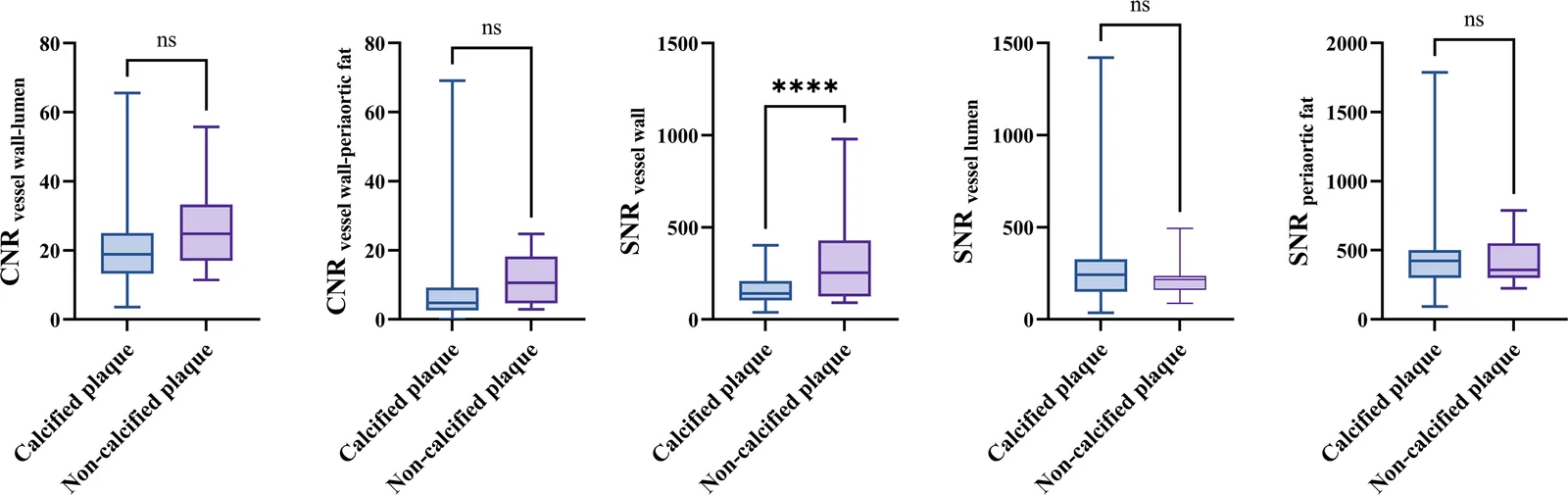
Bar plots show signal-to-noise ratio (SNR) and contrast-to-noise ratio (CNR) among calcified and non-calcified plaque in dark-blood images. The SNR_wall_, SNR_lumen_, SNR_periaortic fat_, CNR_wall-lumen,_ and CNR_wall-periaortic fat_ were respectively analyzed. The ns symbol indicates the value *p*>0.05. The **** symbol indicates the value *p*<0.001.

**Table 1:** Patient characteristics. Data are expressed as mean ± standard deviation or counts (percentage).

**Characteristics**	**Value**
Patient demographics (n = 56)	
Age (years)	65.2 ± 11.4
Male	36 (64.3%)
Body mass index (kg/m^2^)	24.1 ± 3.1
Hypertension	44 (78.6%)
Hyperlipidemia	21 (37.5%)
Diabetes	18 (32.1%)
Plaque characteristics (n =103)	
Calcified	88 (83.8%)
Non-Calcified	15 (14.3%)

**Table 2:** Qualitative image quality scores of dark-blood images. Data are mean ± standard deviation. *p* < 0.05 indicates a statistically significant difference.

	**Image quality score**				***p*-value**		
**Parameter and reader**	**Global segments (n=159)**	**Group A** **Calcified plaque (n=88)**	**Group B** **Non-calcified plaque (n=15)**	**Group C** **Without plaque (n=56)**	**A *vs*. B**	**A *vs*. C**	**B *vs*. C**
Image quality							
Reader 1	4.87±0.39	4.81±0.48	4.93±0.26	4.96±0.19	0.033	<0.001	0.305
Reader 2	4.87±0.43	4.80±0.55	5.00±0	4.96±0.19	0.002	<0.001	0.132
Inner-wall visualization							
Reader 1	4.91±0.33	4.83±0.43	4.93±0.26	5.00±0	0.001	<0.001	<0.001
Reader 2	4.88±0.41	4.80±0.53	5.00±0	4.98±0.13	0.001	<0.001	0.298
Outer-wall visualization							
Reader 1	4.93±0.34	4.89±0.44	5.00±0	5.00±0	0.412	<0.001	<0.001
Reader 2	4.93±0.36	4.89±0.47	4.93±0.26	5.00±0	0.428	<0.001	<0.001

**Table 3:** SNRs and CNRs of dark-blood images and conventional images. Data are mean ± standard deviation. *P* < 0.05 indicates a statistically significant difference.

**Parameter**	**n**	**Quantitative values**		***p*-value**
		**Dark-blood images**	**Conventional images**	
SNR vessel wall				
Global segment	159	174.14±128.16	2.91±2.55	<0.001
Calcified plaque	88	161.92±80.55	3.29±3.16	<0.001
Non-calcified plaque	15	355.02±286.59	2.87±2.14	<0.001
SNR vessel lumen				
Global segment	159	302.20±225.00	34.93±19.96	<0.001
Calcified plaque	88	281.32±217.73	30.84±17.01	<0.001
Non-calcified plaque	15	215.02±93.01	31.66±16.32	0.014
SNR periaortic fat				
Global segment	159	439.34±231.78	12.62±7.11	<0.001
Calcified plaque	88	444.41±258.77	12.51±7.47	<0.001
Non-calcified plaque	15	432.45±159.67	11.98±7.66	<0.001
CNR between the vessel wall and lumen				
Global segment	159	20.89±11.27	10.30±5.91	<0.001
Calcified plaque	88	20.64±11.57	9.51±4.61	<0.001
Non-calcified plaque	15	26.40±13.41	16.73±11.62	0.371
CNR between the vessel wall and periaortic fat				
Global segment	159	7.26±8.93	2.10±2.65	<0.001
Calcified plaque	88	7.24±8.92	2.23±3.23	<0.001
Non-calcified plaque	15	11.85±7.61	2.76±1.63	<0.001

## Data Availability

All the data and supporting information is provided within the article.

## LIST OF ABBREVIATIONS

CAD: Coronary artery disease
CCTA: Coronary computed tomography angiography
SDCT: Dual-layer spectral detector CT
VNC: Virtual non-contrast
VMI: Virtual monoenergetic image
keV: Kilo electron-volt
CTDI_vol_: Volume of CT dose index
DLP: Dose length product
ED: Effective dose
LAD: Left anterior descending
RCA: Right coronary artery
ROI: Region of interest
CPR: Curved planar reconstruction
LCX: Left circumflex artery
SNR: Signal-to-noise ratio
CNR: Contrast-to-noise-ratio
SD: Standard deviation.

## STANDARDS OF REPORTING

STROBE guidelines were followed.
